# Late Onset of Left Atrial Appendage Thrombus Under Oral Anticoagulation in a Patient with a History of Left Atrial Appendage Isolation Using Cryoballoon

**DOI:** 10.19102/icrm.2025.16011

**Published:** 2025-01-15

**Authors:** Cem Çöteli, Samuray Zakariyayev, Ugur Nadir Karakulak, Hikmet Yorgun, Kudret Aytemir

**Affiliations:** 1 Department of Cardiology, Faculty of Medicine, Hacettepe University, Ankara, Turkey; 2 Department of Cardiology, Cardiovascular Research Institute Maastricht (CARIM), Maastricht University Medical Center+, Maastricht, The Netherlands

**Keywords:** Atrial fibrillation, cryoballoon, left atrial appendage closure, left atrial appendage isolation, thrombus formation

## Abstract

Empirical left atrial appendage isolation (eLAAi) using a cryoballoon reduces atrial tachyarrhythmia recurrences in persistent atrial fibrillation. Nonetheless, the most significant concern associated with this procedure is the risk of thromboembolic events, particularly without consistent oral anticoagulant (OAC) use. This case highlights a late thrombus formation post-eLAAi despite proper OAC adherence, raising questions about OAC’s effectiveness in such scenarios. The case suggests considering percutaneous left atrial appendage closure after eLAAi, even in patients with thrombus and ongoing OAC therapy.

## Introduction

Cryoballoon (CB)-based pulmonary vein isolation (PVI) is an effective approach for treating atrial fibrillation (AF).^1,2^ Additionally, empirical left atrial appendage isolation (eLAAi), combined with PVI, may enhance the success of catheter ablation therapy in patients with persistent AF (PeAF).^3,4^ Despite the favorable outcomes of LAA isolation in patients with PeAF, the primary drawback of this approach is the reduction in LAA velocity, which is associated with thromboembolic events.^5^ Thrombus formation following eLAAi can occur shortly after the procedure, and these thromboembolic events are generally linked with the cessation of oral anticoagulation.^6,7^ In this case, we aim to present a patient with a history of eLAAi using a CB who developed an LAA thrombus during very late follow-up despite the uninterrupted use of a therapeutic dosage of oral anticoagulant therapy. Written informed consent was obtained from the patient for this case report. The study has been approved by the local institutional ethics committee.

## Case presentation

**Figure 1:**
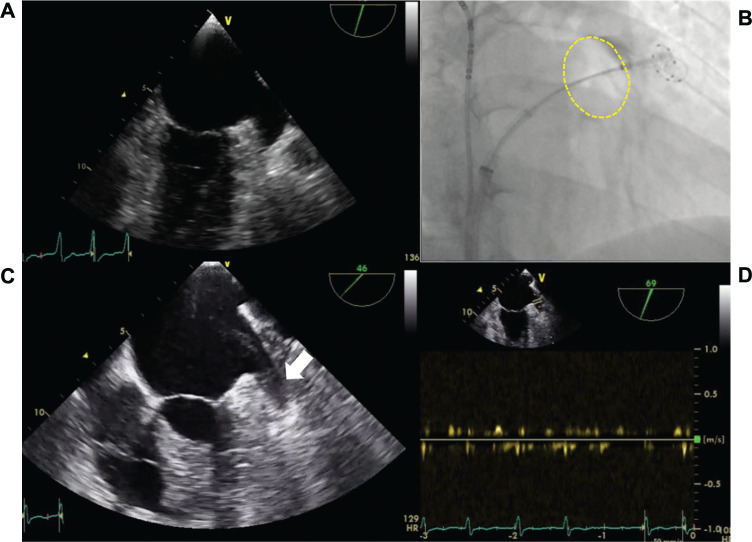
**A:** Preprocedural transesophageal echocardiography (TEE) revealed the absence of thrombus or spontaneous echocontrast (SEC) within the left atrial appendage (LAA). **B:** Successful occlusion of the LAA using a third-generation cryoballoon (yellow dashed contour)—right anterior oblique 30° view. **C:** Postprocedural TEE at the third month indicated grade 2 SEC (white arrow), but there is no evidence of thrombus formation within the LAA. **D:** LAA velocity was measured at 0.24 m/s.

A 67-year-old female patient with symptomatic persistent AF, despite anti-arrhythmic drug therapy, was scheduled for AF ablation. Her medical history revealed a CHA_2_DS_2_-VASc score of 4 points. The left ventricle ejection fraction was 62%, and the left atrial diameter was measured at 44 mm using transthoracic echocardiography. Preprocedural transesophageal echocardiography (TEE) was performed under AF rhythm, and it indicated normal flow within the LAA with a velocity of 0.54 m/s and no evidence of spontaneous echocontrast (SEC) or thrombus formation **(Figure 1A)**.

CB-based PVI and eLAAi were planned as the index procedure for the patient. The patient was referred to the procedure under AF rhythm. After successfully isolating all pulmonary veins (PVs), a circular mapping catheter was positioned into the LAA under fluoroscopy, guided by real-time electrogram recordings. The balloon was then inflated and advanced to the ostium of the LAA. To ensure optimal occlusion, contrast media was administered **(Figure 1B)**. The application was performed for 300 s, as described in detail in our previous paper.^3^ During this procedure, the left phrenic nerve was paced via the left subclavian vein for monitoring. After application, both entrance and exit blocks of the LAA were confirmed. The patient was discharged uneventfully the following day, under a regime of apixaban (5 mg twice daily) and flecainide (100 mg twice daily).

As part of the routine postprocedural follow-up protocol, the patient underwent TEE control in the third month following the procedure. At this time, the patient’s rhythm was AF. TEE indicated a reduction in LAA flow velocity (0.24 m/s) with grade 2 SEC, but there was no evidence of thrombus formation **(Figures 1C and 1D)**. The electrical cardioversion was performed, and the patient was strongly advised to adhere rigorously to oral anticoagulation therapy. The patients experienced another recurrence 12 months after the initial procedure, leading to another cardioversion to restore sinus rhythm.

**Figure 2:**
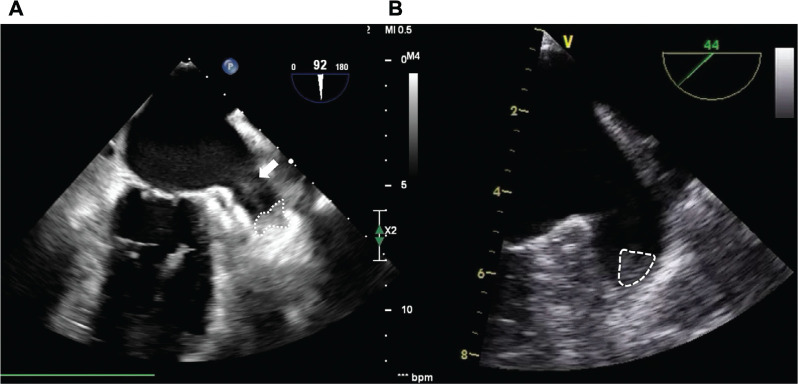
**A:** Forty-four months after empirical left atrial appendage (LAA) isolation, transesophageal echocardiography (TEE) revealed the presence of a thrombus (white dashed contour) within the distal part of the LAA, and spontaneous echocontrast (SEC) in the proximal part of the LAA (white arrow). **B:** Following a 6-week period, a subsequent TEE was performed, and the LAA thrombus remained persistent, while the SEC had resolved (white dashed contour).

Forty-four months after the index procedure, electrical cardioversion was scheduled due to the AF recurrence. However, preprocedural TEE revealed a thrombus in the distal part of the LAA with a flow velocity of 0.38 m/s **(Figure 2A)**. Electrical cardioversion was postponed, and acetylsalicylic acid therapy (100 mg once daily) was initiated in addition to apixaban (5 mg twice daily) to facilitate LAA thrombus resolution. Despite a 6-week oral anticoagulation and antiplatelet therapy combination, LAA thrombus remained persistent. Furthermore, the percutaneous LAA closure procedure was planned for the patient **(Figure 2B)**.

Percutaneous LAA closure was performed using a no-touch technique, as detailed in our previous paper.^8^ Briefly, the transseptal puncture was conducted using the modified Brockenbrough technique. Initially, the sheath was placed in the left superior PV and then pulled back to the ostium of the LAA. Under the guidance of TEE and fluoroscopy, the umbrella part of a 23-mm LAmbre™ (LifeTech Scientific, Shenzhen, China) device was opened at the LAA ostium and gently pushed to the landing zone, trapping the thrombus in the LAA. After achieving optimal settling of the umbrella on the landing zone, the disc was opened at the LAA ostium. Stability was confirmed by gently pulling back the entire system; once stability was ensured, the device was released **(Video 1)**. The patient was discharged the following day without any periprocedural complications, continuing treatment with acetylsalicylic acid (100 mg once daily) and apixaban (5 mg twice daily). There were no additional complications during the 3-month follow-up. Acetylsalicylic acid was discontinued at the 3-month follow-up, but it was planned to continue apixaban 5 mg twice daily for long-term anticoagulant therapy.

**Video 1:** Percutaneous Left Atrial Appendage Closure in Patients with LAA thrombus following LAA Isolation. Watch video here: https://www.dropbox.com/scl/fo/qxcxwlq7bx1ncy6h807od/ADHKjR-klJnD6SskPtwer_g?rlkey=c08l8fds0giw98gdhijefbxec&st=fw7107sq&dl=0

## Discussion

This case report underscores the potential for thrombus formation and thromboembolic complications related to eLAAi, which may manifest after an extended follow-up period. This risk persists even in patients adhering to an uninterrupted therapeutic regimen of oral anticoagulants.

The development of LAA thrombi following eLAAi is acknowledged as a significant complication.^6^ Various factors may predispose thrombus development, including diminished LAA flow velocity, the chronicity of AF, a decreased left ventricular ejection fraction, increased left atrial volume, LAA morphology, and discontinuation of oral anticoagulation therapy.^9–11^ While specific guidelines for post-isolation management of the LAA are not well-established, previous research has indicated a heightened risk of thromboembolic events.^6,7^ Notably, thrombus formation has been documented in patients experiencing ischemic episodes post-eLAAi, predominantly during early follow-up periods.^7^ In this case, we report the occurrence of thrombus formation 44 months after the procedure.

Observational trials also showed that LAA closure is an effective and feasible option following eLAAi.^12,13^ Moreover, a recent expert consensus document has suggested percutaneous LAA closure as a strategy to mitigate ischemic complications post-LAAi, albeit with lower recommendation levels.^14^ Moreover, some centers perform LAA closure in the same procedure as AF ablation; no additional risk has been reported with this approach.^15^ Notwithstanding, a substantial number of patients are maintained on therapeutic anticoagulant regimens. Our case highlights the critical observation that even therapeutic doses of anticoagulants may not suffice to avert thrombus formation in the LAA or prevent thromboembolic incidents. This scenario prompts an essential query: “is there a need for routine LAA closure following eLAAi, even in patients who can adhere to uninterrupted oral anticoagulant therapy (OAC)?”

LAA closure has also emerged as an effective alternative for managing LAA thrombi and diminishing the risk of embolic events.^8^ This case emphasizes the need for consistent follow-up and vigilant monitoring of patients after LAAi. Consequently, it may be prudent to consider TEE in all patients with a history of eLAAi. Furthermore, LAA closure might be advisable, even in patients presenting with LAA thrombus, as a measure to mitigate the heightened risk of thromboembolic events associated with the procedure.
